# Unveiling Preeclampsia Prognosis: Uterine Artery Doppler Indices in Low-Risk Pregnancies

**DOI:** 10.7759/cureus.46060

**Published:** 2023-09-27

**Authors:** Subrat Panda, Vinayak Jante, Ananya Das, Wansallan Shullai, Nalini Sharma, Ritisha Basu, Pratitee Baruah, Makakmayum Ruksana, Namita Gowda

**Affiliations:** 1 Department of Obstetrics and Gynaecology, North Eastern Indira Gandhi Regional Institute of Health and Medical Sciences, Shillong, IND

**Keywords:** ndi, bilateral notching, uterine artery ri, uterine artery pi, preeclampsia prediction

## Abstract

Background

Anticipating preeclampsia’s onset is pivotal in mitigating adverse maternal and perinatal outcomes. This study aims to prognosticate preeclampsia within low-risk pregnancies by evaluating uterine artery Doppler indices within the 14-28 week gestation.

Methodology

An observational cohort comprising 360 low-risk pregnancies (14-28 weeks gestation) underwent serial uterine artery Doppler assessments at 14-20 and 20-28 weeks. Follow-up was extended to delivery to detect preeclampsia incidence.

Results

Among 360 participants, 56 (15.5%) developed preeclampsia. Sensitivity values for resistance index (RI), pulsatility index (PI), and bilateral notching were 17.6%, 56.25%, and 71%, respectively, during 14-20 weeks. Similarly, during 20-28 weeks, sensitivities for RI, PI, and bilateral notching were 16.6%, 36.8%, and 55.5%, respectively, with specificity exceeding 90%. Notch depth index (NDI) >0.14 emerged as a better predictor of preeclampsia between both intervals (area under the curve = 0.686 and 0.646).

Conclusions

Bilateral notching during 14-20 weeks and NDI >0.14 within 14-20 and 20-28 weeks indicate preeclampsia susceptibility in low-risk pregnancies. Conversely, uterine artery Doppler indices at 14-28 weeks effectively rule out preeclampsia development, exhibiting a specificity of >90%.

## Introduction

Hypertensive disorders are the underlying catalysts for complications in 5-10% of pregnancies. Coupled with hemorrhage and infection they form an integral facet of the perilous triad, exerting a substantial impact on maternal well-being and mortality rates [1]. Preeclampsia is a prevalent hypertensive disorder, afflicting approximately 4-7% of pregnancies and emerging as a prominent contributor to maternal mortality. Globally, preeclampsia impacts around 2-5% of expectant mothers, tragically accounting for the loss of approximately 76,000 women and 500,000 infants each year [2]. The incidence and prevalence of preeclampsia and eclampsia in India are notably elevated, with rates at approximately 28% and 7.4-11.3%, respectively, surpassing their global occurrences [3]. In the northeast part of India, the incidence of preeclampsia is 9.7% [4]. In Assam, pregnancy-induced hypertension (PIH) (17.3%) is the major cause of maternal death [5]. Preeclampsia is linked to several common obstetric complications, including intrauterine fetal death; fetal growth restriction; preterm delivery; hemolysis, elevated liver enzymes, low platelet count syndrome; and eclampsia. Furthermore, non-obstetric complications encompass heart failure, peripartum cardiomyopathy, pulmonary edema, heightened susceptibility to future cardiovascular disease, posterior reversible encephalopathy syndrome, stroke, renal failure, acute kidney injury, liver failure, and coagulopathy [6]. Accurate prediction of preeclampsia remains elusive, lacking definitive early pregnancy markers for distinguishing high-risk individuals. Thus, obstetric care primarily centers on early detection; while delivery is the ultimate treatment, proactive measures such as vigilant monitoring and secondary prevention are valuable. Early diagnosis is pivotal for optimized maternal and perinatal outcomes through effective management.

The origin of preeclampsia lies in placental dysfunction, notably impaired uterine spiral artery dilation causing placental ischemia. In healthy pregnancies, controlled trophoblastic invasion remodels spiral arteries, ensuring optimal uteroplacental blood flow. In preeclampsia-prone pregnancies, shallow trophoblastic invasion leaves deep arterioles with retained endothelium, resulting in reduced vessel diameter and blood flow. Early identification of preeclampsia and placental insufficiency is crucial for timely intervention, mitigating maternal and perinatal risks. Our institution lacks serological preeclampsia tests. Maternal uterine artery (UA) Doppler studies have shown superior predictive ability over traditional methods, albeit with variable parameters, protocols, and sensitivity for hypertensive disorders [7].

Predominantly, investigations have concentrated on UA Doppler during the second trimester, aligning with the completion of trophoblastic invasion of maternal spiral arteries. Some scholars have advocated for dual assessment in both the first and second trimesters. Although UA Doppler has gained recognition as a reliable tool for predicting hypertensive disorders in high-risk pregnancies, its utilization within low-risk cohorts remains limited. In regions marked by elevated maternal mortality, where hypertensive disorders wield substantial impact, this study was undertaken within a low-risk demographic. Notably, over half of the women facing such complications lack identifiable risk factors in their history. Employing UA Doppler velocimetry between the 14th and 28th weeks of gestation, this study bears significance.

## Materials and methods

A hospital-based, prospective, observational cohort study was performed in the Department of Obstetrics and Gynaecology, North Eastern Indira Gandhi Regional Institute of Health and Medical Sciences (NEIGRIHMS) for a period of two years (from January 2019 to January 2021). Ethical approval was obtained from the Institutional Ethics Committee of North Eastern Indira Gandhi Regional Institute of Health and Medical Sciences, Meghalaya (approval number: NEIGR/IEC/T8/019). Written and informed consent was taken from each of the participants. A total of 360 pregnant women were included in this study. Preliminary data were collected after obtaining informed written consent from the pregnant patients willing to participate in the study which included thorough history taking to determine the patients’ demographics and gestational age and to assess any high-risk factors associated with the pregnancy. This study enrolled pregnant women with singleton pregnancies, ranging from 14 to 28 weeks of gestation, who sought care at the antenatal clinic. Exclusions encompassed cases of multiple pregnancies, molar pregnancies, established chronic hypertension, ongoing preeclampsia, existing antiphospholipid antibody syndrome during the current pregnancy, instances of fetal congenital anomalies, histories of hypertensive disorders in prior pregnancies, teenage pregnancies, elderly primigravida, diabetes and gestational diabetes, as well as obesity.

Sample size assessment

Clinical assessments included measurements of blood pressure after a 10-minute rest, maternal weight, and fundal height during each visit. Routine urine and blood tests were conducted, with urinary protein estimation performed. UA Doppler recordings were acquired twice: first between 14 and 20 weeks of gestation and then between 20 and 28 weeks. To minimize technical errors, all scans were conducted by a single experienced obstetrician, aided by a skilled radiologist from the Department of Obstetrics and Gynecology at NEIGRIHMS Shillong, utilizing a MINDRAY RESONA7 ultrasound system. Transabdominal ultrasound was used for both UA Doppler examinations.

The procedure involved obtaining a mid-sagittal section of the uterus and visualizing the internal cervical os and cervical canal. The transducer was then gently tilted to identify the right and left uterine arteries using color flow mapping along the uterine cervix’s side, specifically at the internal os level. Pulsed-wave Doppler with a sampling gate set at 3 mm was employed to capture the complete vessel width. The signal was refined until three consistent waveforms were obtained. Mean values were calculated for pulsatility index (PI), resistive index (RI), notch deep index (NDI), and bilateral diastolic notches, recorded between 14-20 weeks and 20-28 weeks of gestation.

Uterine Doppler values were deemed abnormal if RI and PI values exceeded the 95th percentile for the corresponding gestational age, along with bilateral diastolic notches. Standard RI and PI values for the 95th percentile were adopted from prior studies, as well as considering the presence of bilateral diastolic notches.

Statistical analysis

The cutoff value of >0.14 was considered for NDI for both between 14-20 weeks and 20-28 weeks of gestation. When notch was present, the following equation quantified its dept: (D - C/D) to create NDI. The NDI was equal to zero when notch was absent. Qualitative variables were compared using the chi-square/Fisher’s Exact test. Univariate odds ratio along with 95% confidence was calculated to assess the impact of factors on the outcome variable.

Sensitivity, specificity, positive predictive value (PPV), negative predictive value (NPV), and diagnostic accuracy were also calculated. A p-value <0.05 was considered statistically significant. The data were stored in an MS Excel spreadsheet, and statistical analysis was performed using the open-source R programming language.

## Results

In this study, of the 360 women, 56 (15.5%) had preeclampsia while the rest 304 (84.5%) showed no signs of preeclampsia (Table 1).

**Table 1:** Incidence of preeclampsia.

n	Preeclampsia	%
56	Yes	15.5%
304	No	84.5%
360	Total	100%

In this study, the >95th percentile cutoff of RI in both 14-20 weeks and 20-28 weeks was 0.7, and the >95th percentile for PI in 14-20 weeks was 1.8 and 20-28 weeks was 1.5. In 14-20 weeks, PI above the cutoff level was found in 42 cases, and preeclampsia was found in 18 cases. RI above the cutoff level was found in 28 cases, and preeclampsia was found in only six cases. Bilateral diastolic notch was present in 48 cases, out of which preeclampsia was present in only 20 cases. NDI >0.14 was found in 40 cases, and preeclampsia was present only in 18 cases. When PI and RI were combined, 24 women had preeclampsia. When bilateral notch and RI were combined, 12 women had preeclampsia. When bilateral notch and PI were combined, 17 women had Preeclampsia. When PI, RI, and bilateral notch were combined, 19 women had Preeclampsia. On comparing preeclampsia and non-preeclampsia cases, PI and bilateral notch were statistically significant (p = 0.0001 and 0.0001, respectively), but RI was nonsignificant (p = 0.412) (Table 2).

**Table 2:** Incidence of preeclampsia in >95th percentile PI and >95th percentile RI and the presence of bilateral diastolic notch NDI (14-20 weeks). P-value = Pearson’s coefficient; PI = pulsatility index; RI = resistive index; NDI = notch deep index

Preeclampsia		Yes		No		P-value
Uterine artery Doppler (14–20 weeks)		n	%	n	%	
PI	>95th percentile	18	32.14%	24	7.8%	<0.0001
	<95th percentile	38	67.86%	280	92.2%	
RI	>95th percentile	6	10.7%	22	7.2%	<0.412
	<95th percentile	50	89.3%	282	92.7%	
Notch	Present	20	35.8%	28	09.80%	<0.0001
NDI	Absent	36	64.2.%	276	90.19%	0.0001
	>0.14 (40)	18				
	<0.14 (8)	2				
PI + RI (PI/RI)	>95th percentile	24	7%	18	5%	0.0001
	<95th percentile	32	9%	286	79%	
Notch + PI (PI/Notch)	+ve > cutoff	34	9%	14	4%	0.0001
	-ve	22		290		
Notch + RI (Notch/RI)	+ve > cutoff	24	7%	24	7%	0.0001
	-ve	32	9%	280	78%	
Notch + PI + RI (Notch/PI/RI)	+ve > cutoff	38	11%	10	3%	0.0001
	-ve	18	5%	294	82%	

When the area under the curve (AUC) was taken, PI and NDI could be considered good screening tests (0.654 and 0.685, respectively). The sensitivity, specificity, PPV, and NPV for PI of >95th percentile for the development of preeclampsia during 14-20 weeks was 56.25%, 92.6%, 42.8%, and 95.5%, respectively, with a diagnostic accuracy of 89.4%. The sensitivity, specificity, PPV, and NPV for RI of >95th percentile for the development of preeclampsia during 14-20 weeks were 17.64%, 93.2%, 21.4%, and 91.5%, respectively, and diagnostic accuracy was 86.1%. The sensitivity, specificity, PPV, and NPV for bilateral notch for the development of preeclampsia during 14-20 weeks were 71%, 91.5%, 41.6%, and 97.4%, respectively, and the diagnostic accuracy was 90%. When bilateral notch and other diameters such as RI and PI were combined (one, both, or three of them positive), the sensitivity, specificity, PPV, NPV, and diagnostic accuracy increased. The sensitivity, specificity PPV, NPV, and diagnostic accuracy of PI, RI, and bilateral notch were 82.6%, 96.8%, 79.16%, 97.4%, and 95%, respectively. In this study, bilateral notch combined with PI and bilateral notch combined with PI and RI had a reasonable relative risk (10.045 and 13.722, respectively) of developing preeclampsia. The combination of the bilateral notch with PI or RI or both can be used as a screening test as sensitivity combined with specificity was more than 1.5 (Table 3).

**Table 3:** Sensitivity, specificity, PPV, NPV, diagnostic accuracy, and relative risk (14-20 weeks of gestation). PI = pulsatility index; RI = resistive index; PPV = positive predictive value; NPV = negative predictive value; RR = risk ratio

Preeclampsia	Sensitivity	Specificity	PPV	NPV	Diagnostic accuracy	RR
RI	17.64%	93.2%	21.4%	91.5%	86.1%	1.4
PI	56.25%	92.6%	42.8%	95.5%	89.4%	3.58
Notch	71%	91.5%	41.6%	97>4%	90%	3.84
PI + RI	63.1%	94.4%	57.1%	95.5%	91.1%	5.67
Notch + RI	75%	92.6%	50%	97.4%	91.1%	4.87
Notch + PI	80%	95.5%	70.8%	97.4%	93.8%	10.045
Notch + PI + RI	82.6%	96.8%	79.16%	97.4%	95%	13.722

During the 20-28 weeks of screening, 32 women had PI above the cutoff level, but only 14 women developed preeclampsia. In total, 26 women had RI above the cutoff level, and six women developed preeclampsia. Moreover, 40 women had a bilateral diastolic notch, and 20 developed preeclampsia. NDI was >0.14 in 36 women, and 19 developed preeclampsia. When PI and RI were combined, 20 women had Preeclampsia. When notch was combined with RI, 24 women had Preeclampsia. When notch and PI were combined, 24 women had preeclampsia. When PI and RI were combined with a notch, 28 women had preeclampsia. The presence of PI and bilateral notch was statistically significant in preeclampsia and non-preeclampsia women (p = 0.0001 and 0.0001, respectively), but RI was non-significant (0.26). According to the AUC, NDI can be an acceptable test to predict preeclampsia (0.646) (Table 4).

**Table 4:** Incidence of preeclampsia in >95th percentile RI and >95th percentile PI and the presence of bilateral diastolic notch NDI (20-28 weeks). P-value = Pearson’s coefficient; PI = pulsatility index; RI = resistive index; NDI = notch deep index

Preeclampsia		Yes		No		P-value
Uterine artery Doppler (20–28 weeks)		n	%	N	%	
PI	>95th percentile	14	25%	18	5.88%	<0.0001
	<95th percentile	32	75%	286	94.12%	
RI	>95th percentile	6	10.7%	20	7.19%	0.26
	<95th percentile	50	89.3%	284	92.81%	
Bilateral notch	Present	20	35.7%	20	7.19%	<0.0001

The sensitivity, specificity, PPV, and NPV for RI of >95th percentile for the development of preeclampsia during 20-28 weeks were 16.6%, 93.8%, 23.07%, and 91.01%, respectively, and diagnostic accuracy was 86.1%. The sensitivity, specificity, PPV, and NPV for PI of >95th percentile for the development of preeclampsia during 20-28 weeks were 36.8%, 94.4%, 43.75%, and 92.6%, respectively, and diagnostic accuracy was 88.3%. The sensitivity, specificity, PPV, and NPV for the presence of bilateral diastolic notch for the development of preeclampsia at 20-28 weeks were 55.5%, 93.8%, 50%, and 95%, respectively, and diagnostic accuracy was 90%. Similar to 14-20 weeks of gestation, the sensitivity, specificity, and diagnostic accuracy increased when bilateral diastolic notch was combined with PI, RI, or RI combined with notch. The sensitivity, specificity, PPV, and NPV for the presence of bilateral diastolic notch combined with PI and RI (one, both, or three of them positive) for the development of preeclampsia at 20-28 weeks were 63.6%, 96.2%, 70%, and 95%, respectively, and diagnostic accuracy was 95% At 20-28 weeks of gestation, none of the single parameters could be useful as an acceptable predictor of developing preeclampsia. When the bilateral notch was combined with PI or RI, both could be used as an acceptable predictor of preeclampsia (Table 5).

**Table 5:** Sensitivity, specificity, PPV, NPV, diagnostic accuracy, and relative risk (20-28 weeks of gestation). PI = pulsatility index; RI = resistive index; PPV = positive predictive value; NPV = negative predictive value; RR = risk ratio

Preeclampsia	Sensitivity	Specificity	PPV	NPV	Diagnostic accuracy	RR
RI	16.6%	93.8%	23.07%	91.01%	86.1%	1.6
PI	36.8%	94.4%	43.75%	92.6%	88.3%	3.4
Notch	55.5%	93.8%	50%	95%	90%	4.44
PI + RI	45.4%	96.2%	62.5%	92.6%	90%	5.69
Notch + RI	60%	95%	60%	95%	91.1%	6.00
Notch + PI	60%	95.%	60%	95%	93.8%	6.00
Notch + PI + RI	63.6%	96.2%	70%	95%	95%	8.00

## Discussion

Among the cohort of 360 women studied, 56 (15.5%) developed preeclampsia. The prevalence of preeclampsia observed in this study is consistent with Shashi et al.’s findings (20%) and notably higher than the prevalence reported by Scandiuzzi et al. (9.2%) in 2016 and Myatt et al. (6.7%) in 2012. [7-9]. Within the group of 360 women, 48 (13.3%) exhibited notching during the 14-20 week period. The persistence of notching was observed in 11.1% during the 20-28 week interval, comparable to the findings of Myatt et al. (14%). Additionally, Harrington et al. documented preeclampsia in 16% of women displaying bilateral notching at 12-16 weeks of gestation [9,10]. In our study, UA RI values exceeding the 95th percentile were ≥0.70 for both the 14-20 week and 20-28 week periods of pregnancy. These thresholds were derived from prior investigations, such as the study by Scandiuzzi et al., who established 0.77 as the mean RI [7]. In the context of the second trimester, Valensise et al. evidenced that a mean UA RI exceeding 0.58 served as a reliable predictor of hypertensive disorders. [11]. Albaiges et al. evaluated pregnant women at 23 weeks of gestation and obtained a mean UA RI of 0.69 (95th percentile) [12]. Out of the 360 women studied, 40 (11.1%) had an NDI exceeding 0.14, while 88.9% displayed an NDI below this threshold. These outcomes mirror the findings of a study by Ohkuchi et al., indicating a parallel pattern [13]. When considering UA PI values exceeding the 95th percentile for gestational age, 42 women at 14-20 weeks and 32 women at 20-28 weeks exhibited such values. These findings were statistically significant compared to the non-preeclampsia group (p < 0.0001). Sensitivity analysis revealed rates of 56.25% at 14-20 weeks and 36.8% at 20-28 weeks for predicting preeclampsia. These sensitivities are parallel to those of Papageorghiou et al.’s study, which reported a sensitivity of 41% for PI values surpassing the 95th percentile, and Albaiges et al., who observed a sensitivity of 35.3% [14,15]. When evaluating UA RI values exceeding the 95th percentile for gestational age (≥0.70), 28 women at 14-20 weeks and 26 women at 20-28 weeks showcased RI values above this threshold. Importantly, these figures were not statistically significant compared to the non-preeclampsia group (p > 0.05). Sensitivity analysis unveiled rates of 17.64% for both the 14-20 week and 20-28 week intervals in predicting preeclampsia. These detection levels closely align with the outcomes observed in the studies by Bewley et al. and North et al. [16,17]. When considering UA NDI values exceeding 0.14 for gestational age, 40 women at 14-20 weeks and 36 women at 20-28 weeks demonstrated NDI values surpassing this threshold. These findings were statistically significant in comparison to the non-preeclampsia group (p < 0.001). The AUC for NDI was calculated as 0.685 for the 14-20 week interval and 0.646 for the 20-28 week interval. Our study exhibited robust sensitivity for NDI, akin to the findings of the study by Ohkuchi et al. [13. Specifically, the sensitivity was 67%, while the specificity, PPV, and NPV were in alignment with the results of Ohkuchi et al. [13].

Considering only the bilateral UA notch at 14-20 weeks within the cohort of 360 women, 48 displayed this characteristic. Among these, 40 women demonstrated a consistent diastolic notch at 20-28 weeks. The sensitivity, specificity, PPV, and NPV for the presence of bilateral diastolic notch between 14-20 weeks were calculated as 71%, 91.5%, 41.6%, and 97.4%, respectively, yielding a diagnostic accuracy of 90%. At 20-28 weeks, the values were 55.5%, 93.8%, 50%, and 95%, respectively, with a diagnostic accuracy of 90%. Notably, the presence of bilateral diastolic notch held statistically significant importance (p < 0.0001) in distinguishing between preeclampsia and non-preeclampsia groups within this study. Similar outcomes were observed in studies by Pai et al. and Myatt et al. [9,18]. In this study, the sensitivity of combining bilateral notch with RI, bilateral notch with PI, and bilateral notch with both PI and RI was determined to be 75%, 80%, and 82.6%, respectively, during the 14-20 week period. For the 20-28 week interval, these values were 60%, 60%, and 63.8%, respectively. Similar findings were evident in the meta-analysis conducted by Cnossen et al. [19].

To summarize the research findings, at the 14-20 week gestational range, the presence of the bilateral diastolic notch alongside an NDI exceeding 0.14 can be considered as a standalone predictor for preeclampsia. Enhanced prediction is achieved through combined indices, such as bilateral diastolic notch + PI or RI, and PI + RI. However, during the 20-28 week window, no single indicators suffice for preeclampsia prediction. Acceptance is marginally warranted for the combined indices PI + RI + bilateral notching. Throughout both trimesters, all UA Doppler indices are suitable for ruling out preeclampsia development.

However, this study has some limitations, particularly in differentiating between early and late preeclampsia due to limited data, including cases with information gathered via telephonic contact and from deliveries in other institutions.

The broader implication of this research lies in its practical applicability. In settings where serological tests are unavailable but ultrasound expertise is present in outpatient clinics, low-risk pregnant women can be screened during their first-trimester ultrasound. Utilizing UA Doppler indices, including the presence of a bilateral diastolic notch, PI exceeding the 95% cutoff level (1.8), RI surpassing the 95% cutoff level (0.7), and NDI surpassing 0.14, could serve as indicators for predicting preeclampsia. These indicators can be employed during both trimesters to exclude the likelihood of future preeclampsia, given the study’s high specificity results.

## Conclusions

Preeclampsia is an intricate clinical syndrome impacting multiple organ systems and bearing substantial consequences for maternal and perinatal mortality and morbidity. UA indices that signify resistance within the uteroplacental circulation, namely, PI, bilateral notching, and NDI >0.14, display notable elevations in preeclampsia cases. This suggests that the resistance to blood flow holds greater significance than the sheer blood flow volume. UA indices such as PI and NDI emerge as superior predictors for preeclampsia development within low-risk pregnancies, particularly during the 14-20 week gestational window. Furthermore, in both the first and second trimesters, these indices exhibit the potential for ruling out the likelihood of preeclampsia development, as evidenced by significantly elevated specificity and NPV.
